# Spatial Environmental Heterogeneity Determines Young Biofilm Assemblages on Microplastics in Baltic Sea Mesocosms

**DOI:** 10.3389/fmicb.2019.01665

**Published:** 2019-08-09

**Authors:** Katharina Kesy, Sonja Oberbeckmann, Bernd Kreikemeyer, Matthias Labrenz

**Affiliations:** ^1^Biological Oceanography, Leibniz Institute for Baltic Sea Research Warnemünde (IOW), Rostock, Germany; ^2^Institute of Medical Microbiology, Virology and Hygiene, University Medical Center Rostock, Rostock, Germany

**Keywords:** biofilms, microplastics, *Sphingomonadaceae*, *Vibrio*, Baltic Sea, salinity gradient

## Abstract

Microplastics in aquatic environments provide novel habitats for surface-colonizing microorganisms. Given the continuing debate on whether substrate-specific properties or environmental factors prevail in shaping biofilm assemblages on microplastics, we examined the influence of substrate vs. spatial factors in the development of bacterial assemblages on polyethylene (PE), polystyrene (PS), wood, and seston and in the free-living fraction. Further, the selective colonization of microplastics by potential pathogens was investigated because among the bacterial species found in microplastic-associated biofilms are potentially pathogenic *Vibrio* spp. Due to their persistence and great dispersal potential, microplastics could act as vectors for these potential pathogens and for biofilm assemblages in general. Incubation experiments with these substrates were conducted for 7 days during a summer cruise along the eastern Baltic Sea coastline in waters covering a salinity gradient of 4.5–9 PSU. Bacterial assemblages were analyzed using 16S rRNA-gene amplicon sequencing, distance-based redundancy analyses, and the linear discriminant analysis effect size method to identify taxa that were significantly more abundant on the plastics. The results showed that the sample type was the most important factor structuring bacterial assemblages overall. Surface properties were less significant in differentiating attached biofilms on PE, PS, and wood; instead, environmental factors, mainly salinity, prevailed. A potential role for inorganic-nutrient limitations in surface-specific attachment was identified as well. *Alphaproteobacteria* (*Sphingomonadaceae*, *Devosiaceae*, and *Rhodobacteraceae*) and *Gammaproteobacteria* (*Alteromonadaceae* and *Pseudomonas*) were distinctive for the PE- and PS-associated biofilms. *Vibrio* was more abundant on the PE and PS biofilms than on seston, but its abundances were highest on wood and positively correlated with salinity. These results corroborate earlier findings that microplastics constitute a habitat for biofilm-forming microorganisms distinct from seston, but less from wood. In contrast to earlier reports of low *Vibrio* numbers on microplastics, these results also suggest that vibrios are early colonizers of surfaces in general. Spatial as well as temporal dynamics should therefore be considered when assessing the potential of microplastics to serve as vectors for bacterial assemblages and putative pathogens, as these parameters are major drivers of biofilm diversity.

## Introduction

Microplastics, usually defined as plastic particles ≤5 mm in size (Arthur et al., 2009), are now widely recognized as new, significant pollutants of aquatic systems (GESAMP, 2015). Although the first records of microplastics in aquatic systems date back to the 1970s (Carpenter and Smith, 1972), most research into the global pollution of aquatic systems with microplastics has been conducted only within the last 15 years (Thompson R.C. et al., 2004). The majority of these investigations have focused on the potential harm to aquatic organisms resulting from the ingestion of microplastics. Among the effects identified thus far are inflammatory responses in the tissue of the blue mussel *Mytilus edulis* (Browne et al., 2008), reproductive disruption in the Pacific oyster *Crassostrea gigas* (Sussarellu et al., 2016), a reduction in carbon uptake by the copepod *Calanus helgolandicus* (Cole et al., 2015), and reduced growth rates of the cold-water coral *Lophelia pertusa* (Chapron et al., 2018). However, the role of microplastics as a habitat for biofilm-forming microorganisms has only recently been investigated, although interest in this topic is growing (Ivar do Sul et al., 2018).

In aqueous systems, biofilms inevitably form on every submerged surface. Initially, a so-called conditioning film develops in which polysaccharides, amino acids, and proteins immediately adsorb onto the surface and promote subsequent colonization by microorganisms (ZoBell, 1943). Microorganisms are key drivers of all biochemical cycles (Falkowski et al., 2008) and the biofilms that form on surfaces have been shown to host distinct microbial communities with distinct functional traits (Dang and Lovell, 2016). In addition to enhancing microbial activity (van Loosdrecht et al., 1990), biofilms protect microorganisms from environmental stressors, such as UV-radiation, osmotic stress, and antibiotics. Moreover, they provide opportunities for new niches, through versatile metabolic cooperation and horizontal gene transfer (Davey and O’Toole, 2000).

It has been estimated that >5 trillion plastic pieces are afloat at sea, accumulating in ocean convergence zones such as the northern and southern subtropical gyres (Eriksen et al., 2014). The impacts of this vast addition of newly available surfaces colonizable by biofilm-forming microorganisms on aquatic microbial communities and ecosystem functioning have yet to be fully determined. Studies from different regions of the world’s oceans have shown that microbial assemblages on microplastics usually differ from their free-living counterpart and from assemblages on natural seston (Zettler et al., 2013; Dussud et al., 2018b; Oberbeckmann et al., 2018). However, whether biofilm communities are predominantly shaped by environmental factors or surface properties is unclear and the environmental factors exerting the strongest selective pressure have yet to be identified. Oberbeckmann et al. (2014, 2016) found that the microbial assemblages on polyethylene terephthalate (PET) bottles and glass slides incubated in the North Sea for 6 weeks were shaped mainly by seasonal and geographic factors rather than by surface properties. Ogonowski et al. (2018) identified a strong separation between the composition of the bacterial communities on artificial and hydrophobic polymers on the one hand and hydrophilic glass and cellulose substrates on the other after 14 days of colonization. Amaral-Zettler et al. (2015) reported that microplastic-associated assemblages sampled from the Pacific and Atlantic oceans exhibited biogeographic patterns but only a weak relationship with the polymer type. De Tender et al. (2015) assumed that salinity, temperature, and oxygen levels played a role in shaping the microplastic-associated assemblages obtained from sediments. Further, it could be shown that surface properties are more important under low nutrient conditions (Oberbeckmann et al., 2018). However, Dussud et al. (2018b) could not detect an effect of geographic location, environmental factors, or different polymers on the microbial assemblages that had formed on plastics sampled in the western Mediterranean basin. There was also no effect of polymer type or sampling location on the biofilms of microplastic samples obtained from the northern Pacific Ocean (Bryant et al., 2016).

Biofilms can also serve as reservoirs for potentially pathogenic bacteria (Lyons et al., 2010). Shikuma and Hadfield (2010) found that *Vibrio*, a genus which includes potential human pathogens, was enriched in the biofilms on ship hulls compared to the surrounding water in different ports of Hawai’i, United States. Islam et al. (2007) detected *Vibrio cholerae* in biofilms on acrylic glass submerged in a canal in Bangladesh. *Vibrio* spp. were found at high relative abundance (24%) on a polypropylene particle sampled from the North Atlantic Gyre (Zettler et al., 2013), on samples from the Bay of Brest, France (1.5–18.6%) (Frère et al., 2018) and the potential pathogen *V. parahaemolyticus* was identified on microplastic particles sampled from the North Sea and Baltic Sea (Kirstein et al., 2016). However, other studies of microplastic-associated microbial assemblages found little or no enrichment of potential pathogens sampled *in situ* (Schmidt et al., 2014; Dussud et al., 2018b), or after passage through the gut of marine invertebrates (Kesy et al., 2016, 2017). Thus, whether microplastics *per se* selectively favor the colonization of potential pathogens such as *Vibrio* or even become enriched and thus able to serve as vectors for potentially pathogenic bacteria (Oberbeckmann et al., 2018) remains to be determined.

Because of the large volumes of plastic pollutants <5 mm in size (Moret-Ferguson et al., 2010; Cozar et al., 2014) and their persistence in aquatic systems, microplastics could provide a significant route of pathogen dispersal (Pham et al., 2012). Although sediments in the Baltic Sea have been shown to act as reservoirs of *Vibrio* spp. (Huehn et al., 2014), floating microplastics, and thus their attached microbial assemblages, are more susceptible to distribution by winds and currents (Chubarenko et al., 2016) and may therefore be rapidly transported over long distances (Isobe et al., 2014). Furthermore, the microplastics sampled *in situ* are of unknown age and the attached bacterial assemblages have been shown to change over time (De Tender et al., 2017; Dussud et al., 2018a). Studies of biofilm formation must therefore be conducted under controlled conditions in addition to *in situ* investigations, to augment the knowledge on drivers of biofilm diversity and interactions with potential pathogens within the different aquatic habitats.

The Baltic Sea is a semi-enclosed sea in Northern Europe that is under strong anthropogenic pressure (HELCOM, 2010). It has a stable salinity gradient, with nearly marine conditions in its most western regions and nearly freshwater conditions in the northeast. Brackish waters are a suitable habitat for several *Vibrio* species, including the potential human pathogens *V. vulnificus*, *V. cholerae* non-O1, and *V. parahaemolyticus*, which can cause severe wound infections and gastroenteritis (Baker-Austin et al., 2010). Because *Vibrio* infections have been repeatedly reported from the Baltic Sea (Baker-Austin et al., 2013), it is a suitable ecosystem to investigate the influence of different environmental factors on biofilm formation on microplastics, including the colonization of those biofilms by potentially pathogenic *Vibrio*. In this study, we investigated the influence of geographic location vs. habitat type on bacterial assemblages, with a focus on developing biofilm assemblages on two different polymers, and whether potential pathogens are selectively enriched on microplastics. Thus, incubation experiments using polyethylene (PE) and polystyrene (PS) microplastics were conducted. Wood particles served as a biotic control, because their properties are similar to those of floating plastics in terms of elemental structure and floating behavior. The incubations were conducted for 7 days during a cruise along ∼2000 km of the southeastern coastline of the Baltic Sea, covering a salinity gradient of 4.5–9 PSU. The biofilms that developed during those 7 days can still be considered as young, which has been shown in several studies (Fischer et al., 2012; De Tender et al., 2017; Dussud et al., 2018a). Bacterial assemblages were analyzed using 16S rRNA-gene amplicon sequencing, multiple regressions, and linear discriminant effect size to distinguish the effects of sampling station vs. sample type. The colonization and potential enrichment of the particles by putative pathogenic *Vibrio* spp. were assessed by comparing the relative abundances of *Vibrio* spp. on the different sample types.

## Materials and Methods

### Sampling Campaign and Incubation Experiments

Incubation experiments were carried out similar to those described by Ogonowski et al. (2018) during a cruise in August/September 2015 along the coast of the Baltic Sea with the *R/V Poseidon* (cruise POS488), covering roughly 2000 km of coastline along the eastern mesohalinic part of the Baltic Sea, from Rostock, Germany to Helsinki, Finland.

**FIGURE 1:**
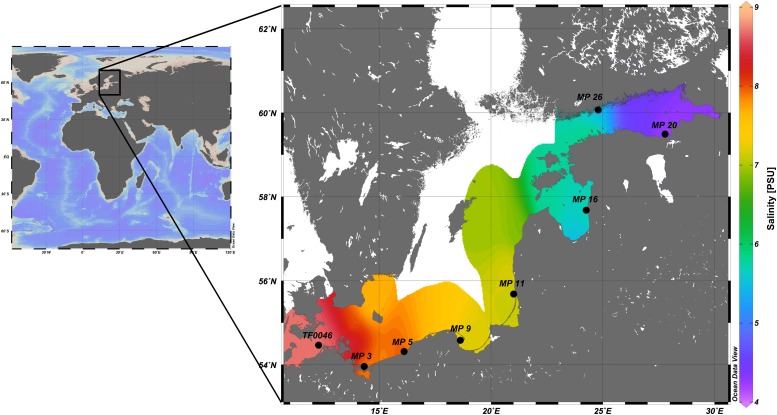
Map of the study location of the Baltic Sea and of the sampling stations included in the incubation experiments along the Baltic Sea coast (enlarged). Salinities in the surface water are those measured during cruise POS488 (some stations are not depicted) and subsequently extrapolated. The map was created using Ocean Data View v. 5.0 (Schlitzer, 2018).

Surface water from within the first 5 m depth was collected at eight stations (Figure 1 and Supplementary Table 1) with 5-L free-flow bottles mounted on a rosette equipped with a conductivity–temperature–depth–probe (Sea-Bird SBE 9). Water from five to six bottles was mixed and then sequentially filtered in technical triplicates (500 mL each) over cellulose nitrate filters (GE Whatman) of 3- (seston-attached bacteria) and 0.22-μm (free-living fraction) pore-size. The bacteria on these filters represented the *in situ* samples (*t*_0_). The filters were snap-frozen in liquid nitrogen and stored at −80°C. The remaining water was then filtered through 30-μm gauze to exclude large grazers and 1.5-L distributed into plastic tanks (SAVIC, 19.5 × 13 × 11.5 cm). The plastic and wood as control substrates used were the same as described in Oberbeckmann et al. (2018). For the treatment incubation, 80-resin PE pellets (HDPE HTA108, ExxonMobil, density 0.961 g cm^–3^), 80-resin PS pellets (polystyrene 143 E, BASF, density 1.04 g cm^–3^; both ø 3 mm, respectively), and 2-g wood pellets (1Heiz^®^, Germany) were introduced together into the treatment tanks (treatment incubation, *n* = 3, Supplementary Figure 1). Tanks containing only water, without plastic or wood particles, served as the control (control incubation, *n* = 3, Supplementary Figure 1). The treatment and control incubations were run for 7 days at the ambient temperature (18–20°C) and were aerated using common aquaria diffuser stones (Dohse Aquaristik, Germany). Light/dark cycles varied between 19/5 and 18/6 h. Prior to the experiment, all materials used in the study were incubated in Milli Q Water (Merck Millipore) for at least 24 h, to allow the leaching out of any additives from the material, and then dried at 30°C. Temperature, O_2_, salinity, and the pH of the incubation water were monitored during the course of the experiment using a HachLange field meter and ready-to-use pH-indicator strips (Merck, Germany).

After 7 days, the PE, PS, and wood particles were collected using sterile tweezers, rinsed twice with sterile-filtered seawater, and quickly centrifuged to remove loosely attached cells. The remaining water was removed and the particles were snap-frozen. To assess the bacterial assemblages on seston and in the free-living fraction of the incubations at *t*_7_, water (500 mL) from all incubations was pre-filtered over a 100-μm gauze. This step was necessary to exclude smaller wood particles. The pre-filtered water was then processed as described for the *in situ* samples. All samples were stored at −80°C until further analysis.

Additionally, 40 mL of water from each tank was collected, filtered through an Acrodisc 0.2 μm HT Tuffryn Membrane Syringe Filter (PALL Life Science) to remove any particles and stored at −20°C for later nutrient analysis. Nutrient analysis for the *in situ* samples was performed on board, using standard colorimetric methods (Grasshoff et al., 1999), and for the *t*_7_ samples, after the cruise, using an autoanalyzer (Seal Analytical). Because ammonia concentrations cannot be measured reliably after freezing and subsequent thawing of samples, they were omitted from the *t*_7_ dataset.

### DNA Extraction and 16S rRNA-Gene Amplicon Sequencing

DNA was extracted from all sample types using the DNeasy PowerSoil Kit (Qiagen) according to the manufacturer’s instruction, except that DNA was eluted twice from the spin column, using the same 50 μL of PCR-grade water, to enhance the DNA yield. Twelve PE and PS pellets and 45 mg of wood were used for each DNA extraction. Blank extractions were carried out after each extraction kit package had been used, to account for possible contamination during the extraction process (Salter et al., 2014). The DNA was PCR-amplified using primers covering the V4 region of the 16S rRNA-gene (position 515F–806R), with the forward sequence 5′-GTGCCAGCMGCCGCGGTAA-3′ and the reverse sequence 5′-GGACTACHVGGGTWTCTAAT-3′ (Caporaso et al., 2011). The PCR was preceded by a short linear amplification step to increase the DNA yield. Thermal cycling started with an initial denaturation at 98°C for 2 min, followed by an additional denaturation step at 98°C for 15 s, annealing at 65°C for 15 s, and elongation for 30 s at 68°C. The last three steps were repeated nine times, with the elongation temperature reduced by 1°C per cycle (linear amplification), followed by a denaturation step at 98°C for 15 s, annealing at 55°C for 15 s, and an elongation step at 68°C for 30 s (24 cycles). Thermocycling ended with a final elongation step at 70°C for 5 min (Takahashi et al., 2014). Library preparation and sequencing on an Illumina MiSeq machine were carried out according to the “Illumina 16S Metagenomic Sequencing Library Preparation Guide.” DNA from a known *V. vulnificus* strain (DSM No. 10143^*T*^) and PCR-grade water were included in each run to serve as a positive and negative control, respectively.

### Sequence Processing

Raw sequence reads were processed using the mothur pipeline v. 1.39.5 (Schloss et al., 2009) following the mothur MiSeq SOP guidelines (Kozich et al., 2013; MiSeq SOP, 2018). Quality filtered sequences were classified using the k-Nearest Neighbor algorithm and the SILVA SSURef release 132 as the reference database (Yilmaz et al., 2014), with a required bootstrap of ≥85%. The taxonomy used in the 132 release and throughout this study incorporated several rearrangements of bacterial phyla, as proposed by Parks et al. (2018). Operational taxonomic units (OTUs) were clustered based on 97% sequence similarity and those with sequence reads ≤3 in the whole dataset were excluded. Sequences classified as Mitochondria, *Archaea*, Chloroplasts, and *Eukaryota* were also excluded.

The dataset was further filtered so that OTUs with mean read counts of 2.5 in the blank extraction or in the negative controls were discarded. The maximum library size of the PE, PS, and wood pellets incubated in Milli Q water was 203 reads after filtering; these samples were therefore omitted from the dataset.

The raw sequences obtained in this study were deposited in the NCBI Sequence Read Archive (SRA) under the accession number PRJNA506548.

### Chao1 Richness and Species Turnover

For the α- and β-diversity analyses, the filtered dataset was subsampled to the smallest library size (13,926 sequences) using 100 iterations, and the mean reads per sample and per OTU were calculated (Zha et al., 2016) together with the mean OTU richness based on the Chao1 estimator and Pielou’s evenness. The Kruskal–Wallis test was used to determine whether the Chao1 richness and Pielou’s evenness were significantly different between sample types and between stations. If the results of the Kruskal–Wallis test were statistically significant, *post hoc* pair-wise comparisons were performed using the Conover–Iman test for multiple comparisons within the conover.test package v. 1.1.5 in R (Dinno, 2017). A Benjamini–Hochberg correction was applied to *p*-values for multiple testing. The results were considered significant at an α-level of 0.05. A Venn diagram was computed using the package VennDiagram in R (Chen, 2018) to assess the number of unique OTUs within each sample type. All *t*_0_ and *t*_7_ samples from the seston and from the free-living fraction were combined, respectively, prior to computing of the Venn diagram to account for OTUs truly unique to the plastics.

### Relative Abundances of the Most Abundant Bacterial Classes

Relative abundances were calculated within mothur using the “get.relabund” command and transformed to a percentage in the R program (R Core Team, 2017). Relative abundances at the class level were visualized for classes with a mean relative abundance of ≥1% in a least one sample, using the ggplot2 package (Wickham, 2016).

### Plastic-Specific Bacteria

To evaluate OTUs that discriminated between sample types, the linear discriminant analysis effect size method (LEfSe; Segata et al., 2011) was applied to the relative-abundance-based OTU table of the filtered dataset. Default parameter settings were used and an all-against-all comparison strategy was applied. First, the PE, PS, and wood samples were combined into a single group to determine whether a core community was present on the introduced particles. In a second LEfSe run, only the PE and PS samples were combined, yielding a plastics group, to evaluate OTUs that were significantly more abundant on plastics than on wood or seston or in the free-living fraction. The core OTUs of the combined PE, PS, and wood samples as well as the discriminant OTUs for the wood and plastics samples alone were visualized at the family level in a phylogenetic tree constructed from all OTUs with a mean relative abundance of ≥0.1% in at least one sample type. The relaxed neighbor-joining method contained in the clearcut program within mothur (Evans et al., 2006) was used and the tree was visualized using the interactive Tree Of Life online tool (iTOL, v. 4.2.3; Letunic and Bork, 2016).

### *Vibrio* spp. Relative Abundances

To evaluate the proportion of *Vibrio* spp. within the total bacterial assemblages, the mean relative abundances of each *Vibrio* OTU and the standard deviation per triplicates were calculated in R and visualized using the ggplot2 package. The Kruskal–Wallis test and Conover–Iman test for pair-wise comparisons were used to identify significant differences in the relative abundances on seston and in the free-living fraction between the treatment incubations, control incubations, and the *in situ* samples. The same tests were applied to determine differences between all sample types within the treatment and control incubations. Because only two replicates were available for the seston samples from the treatment incubations of station MP9, these comparisons were excluded, when applicable. A Spearman rank correlation (ρ) was used to correlate *Vibrio* spp. read counts to environmental parameters.

### Multiple Regression Analysis of Factors Influencing Bacterial Assemblages

Data in the read-based, subsampled OTU table of the *t*_7_ samples, as described for the Chao1 richness, were further square-root-transformed and used for all multiple regression analyses and multivariate statistics. To test whether the bacterial assemblages differed significantly from each other, global and pair-wise permutational multivariate analyses of variance (PERMANOVAs; Anderson, 2001) were calculated on the Bray–Curtis similarity matrix for a two-factorial design (sample type and station). Pair-wise comparisons were calculated for the factor “sample type” within each station to exclude possible effects between stations, using Monte Carlo random draws from the asymptotic permutation distribution (Anderson and Robinson, 2003). To account for possible dispersal effects between samples, the homogeneity of the dispersions was tested using the PERMDISP routine (Anderson, 2006). To determine whether substrate type or geographic location was the main driver of the bacterial assemblages, a distance-based redundancy analysis (dbRDA; Legendre and Anderson, 1999) was performed based on the Bray–Curtis dissimilarity matrix, using the sample types (PE, PS, wood, seston, free-living) and the different stations as constraining factors. The dbRDA was conducted in R using the “dbrda” function from the vegan package (Oksanen et al., 2018), with Lingoes correction for negative eigenvalues (Lingoes, 1971). Significance tests of the dbRDA models and marginal tests for the factors were performed using permutation tests with the “anova.cca” function of the vegan package (999 permutations). All regression coefficients (*R*^2^) were adjusted for multiple testing. The contributions of constraining factors to the first two axes of the dbRDA model were assessed with Spearman rank correlations (ρ) using the basic “cor” function in R. Because of missing water samples for the station MP5 incubations, data from this station were excluded from the dbRDA and the PERMANOVA during comparisons of all sample types. The “ordisurf” function from the vegan package was used to fit the response surfaces of salinity, temperature, ${\text{NO}}_{2}^{-}$, ${\text{NO}}_{3}^{-}$, and ${\text{PO}}_{4}^{3-}$ (means between *t*_0_ and *t*_7_) onto the dbRDA plots (Bennion et al., 2012).

All tests were performed in the R program for Statistical Computation v. 3.4.3 (R Core Team, 2017) using the packages vegan v. 2.4-6 (Oksanen et al., 2018), reshape2 v. 1.4.3 for data handling (Wickham, 2007), and ggplot2 v. 3.0.0 for visualization (Wickham, 2016). Graphs were further processed with Inkscape v. 92.0. PERMANOVA and PERMDISP tests were performed using the PRIMER7 program and its add-on package PERMANOVA+ (PRIMER-e, Quest Research Limited, Auckland, New Zealand).

## Results

### Physico-Chemical Parameters of the Stations and Inorganic Nutrient Concentrations Over the Course of the Experiment

The *in situ* salinity of the experimental stations ranged from 8.7 PSU at the most western station (TF0046) to 4.4 PSU at the most eastern station (MP20) (Figure 1). The temperature of the surface waters was consistent between 18.5 and 20.6°C, except at stations TF0046 (15.3°C) and MP5 (10.0°C).

Inorganic nitrogen (${\text{NO}}_{2}^{-}$, ${\text{NO}}_{3}^{-}$, and ${\text{NH}}_{4}^{+}$) was depleted at all stations (<0.5 μmol L^–1^), except at station MP16 (2.1 μmol L^–1^). Phosphate concentrations ranged between 0.05 μmol L^–1^ at station MP20 and 0.66 μmol L^–1^ at station MP5. Most stations were therefore extremely nitrogen-limited, with DIN/DIP ratios <2.2, except stations MP20 (DIN/DIP 17.8) and MP16 (DIN/DIP 29.8), which were rather phosphate-limited (Supplementary Table 1).

At the end of the incubation experiments, salinity had increased slightly, to between 0.3 and 0.8 PSU, due to evaporation, and the temperature was the same in all incubation tanks, between 18 and 20°C (Supplementary Table 1). Dissolved inorganic nitrogen (DIN) concentrations (without ${\text{NH}}_{4}^{+}$) were still very low (<0.3–0.6 μmol L^–1^), except in the incubation tanks of station MP11 (1.7 ± 1.5 μmol L^–1^), while phosphate was nearly depleted in the incubation tanks of all stations, with concentrations <0.1 μmol L^–1^ (Supplementary Table 1). Due to the low DIN concentrations, the incubations continued to be nitrogen-limited (DIN/DIP 2.5–6.1), except at station MP11 (DIN/DIP 18.6; Supplementary Table 1).

### Sequence Yield and Quality

The four Illumina MiSeq runs generated 38,024,360 paired-end reads. Assembly of the forward and reverse reads yielded 33,166,861 sequences. The final sequence count after filtering was 14,199,783. Based on a 97% similarity, these sequences could be clustered into 12,572 OTUs. After the removal of potential contaminating OTUs, which were also found in the negative controls and blank extractions, 12,509 OTUs remained in the whole dataset.

### Bacterial Richness on Different Sample Types and Across Stations

**FIGURE 2:**
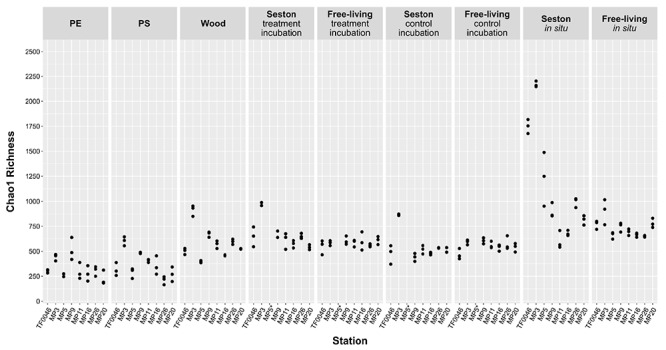
Chao1 estimator of bacterial OTU richness on seston (≥3 μm) and in the free-living fraction (3–0.22 μm) at different stations at *t*_0_ (*in situ*) and after 7 days of incubation on PE, PS, wood, and seston, and in the free-living fraction. Data for both the treatment and control incubations are shown. ^*^For station MP5, incubation water samples were not available.

Chao1 richness differed according to sample type, but also according to the station (Figure 2) and mean richness across all stations was significantly lower on PE and PS than on wood or seston and compared to the free-living fraction (*p* < 0.001, Supplementary Table 2). The mean Chao1 richness across all stations on PE and the PS was 329 ± 108 and 366 ± 130, respectively. In the treatment and control incubations, the mean Chao1 richness across all stations on wood and seston and in the free-living fraction was relatively similar, ranging from 667 ± 148 on seston from the treatment incubations to 579 ± 154 on wood and 554 ± 56 in the free-living fraction of the control incubations, although some significant differences were detected (Figure 2 and Supplementary Table 2). Mean Chao1 richness was significantly higher in the *in situ* samples of seston and the free-living fraction (1142 ± 534 and 733 ± 94; *p* < 0.001 and *p* ≤ 0.007, respectively; Supplementary Table 2). In addition, for all sample types, except those of the free-living fraction of the treatment and control incubations, the differences in Chao1 richness between stations were significant (*p* = 0.003–0.05, Supplementary Table 2). Mean Chao1 richness across all sample types was generally highest at station MP3: 894 ± 496, except in the cases of PE and the free-living fraction of the treatment and control incubations (Figure 2).

Pielou’s evenness was relatively uniform between sample types across all the stations, ranging from 0.62 ± 0.05 on the seston samples of the control incubation to 0.74 ± 0.01 in the free-living fraction *in situ* (Supplementary Figure 2). Although both factors, “sample type” and “station,” had a significant effect on evenness (*p* < 0.001 and 0.013, respectively), there was no obvious pattern between sample types and stations (Supplementary Figure 2). However, evenness was lowest on the PE and PS samples at the western stations TF0046, MP3, and MP5, ranging from 0.6 ± 0.02 for the PE samples at station TF0046 to 0.68 ± 0.01 for the PS samples at station MP3 (Supplementary Figure 2).

### β-Diversity

The lowest number of unique OTUs was associated with PE, PS, and wood (50, 93, and 137 OTUs, respectively). These three sample types had 20 OTUs in common and 100 OTUs that were shared with seston. The latter had the highest number of unique OTUs (3184), followed by the free-living fraction (1772 OTUs). Among all sample types there were 1098 shared OTUs. There was also a pronounced overlap of OTUs shared by seston and wood (603 OTUs), by seston, wood, and the free-living fraction (670 OTUs) and by seston and the free-living fraction (1969 OTUs, Supplementary Figure 3).

### General Community Composition on Class Level

**FIGURE 3:**
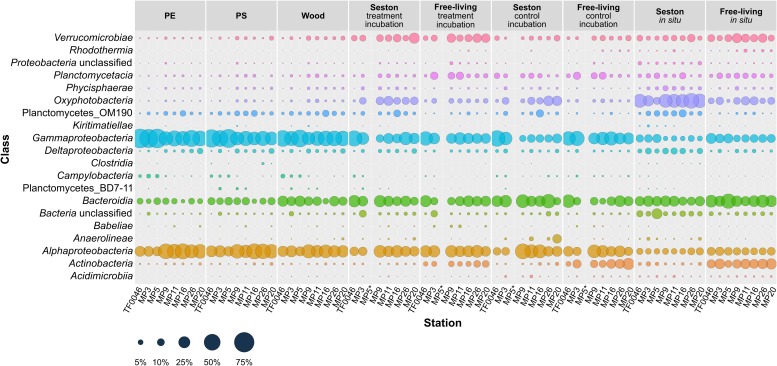
Mean relative abundances of the bacterial classes (>1% relative abundance in at least one sample) present on seston (≥3 μm) and in the free-living fraction (3–0.22 μm) at different stations at *t*_0_ (*in situ*) and in the samples incubated for 7 days on PE, PS, wood, and seston and in the free-living fraction. Data for both the treatment and the control incubation are shown. ^*^For station MP5, incubation water samples were not available.

*Gammaproteobacteria*, *Alphaproteobacteria*, and *Bacteroidia* were the most abundant classes overall. Twenty classes of 12 phyla occurred in abundances of ≥1% in at least one sample. Some classes were found in larger quantities on PE, PS, and wood and some also differed in their occurrences depending on the station (Figure 3).

*Gammaproteobacteria* was the dominant class in samples from the treatment and control incubations, with greater mean abundances across all stations on PE (52.1 ± 13.5%), PS (43.9 ± 12.4%), and wood (42.6 ± 7.5%) than on seston (24.9 ± 11.3 in the treatment and 20.0 ± 14.6% in the control incubations) or in the free-living fraction (23.5 ± 6.3 and 30.7 ± 6.8%, respectively) of the incubation waters. The mean abundance of *Gammaproteobacteria* was less on the seston *in situ* samples and in the *in situ* samples of the free-living fraction (7.9 ± 3.7 and 13.7 ± 3.4%, respectively). In addition, the relative abundance of *Gammaproteobacteria* differed depending on the location and was greater at the western stations TF0046, MP3, and MP5 (maximum abundance of 68.8 ± 0.6% on PE and 63.9 ± 2.4% on PS at station MP5). The lowest percentage on PE occurred at station MP9 (35.4 ± 1.4%) and on PS at station MP16 (32.4 ± 4.0%) (Figure 3).

The second most abundant class was *Alphaproteobacteria*, which was also generally found in higher mean numbers in the incubations than in the *in situ* samples across all stations. *Alphaproteobacteria* were also slightly more abundant on the plastics (31.6 ± 11.5% on PE and 32.1 ± 9.4% on PS) than on wood (26.0 ± 5.7%) or seston (20.5 ± 5.4% in the treatment and 24.7 ± 12.3% in the control incubations) or in the free-living fraction (24.3 ± 3.0% in the treatment and 21.1 ± 6.7% in the control incubations). Low abundances of *Alphaproteobacteria* also characterized the *in situ* samples: 7.7 ± 1.6% on seston and 11.4 ± 1.5% in the free-living fraction. In the treatment and control incubations, the relative abundances of *Alphaproteobacteria* showed a general trend toward higher percentages at the more eastern stations (MP9–MP26). The maximum abundances on PE and PS were measured at station MP16 (44.7 ± 3.5 and 44.6 ± 2.7%, respectively), while the lowest abundance occurred at station MP5 (16.2 ± 2.0 and 18.0 ± 1.0%, respectively). This trend was not observed in the *in situ* samples (Figure 3).

The occurrence of uncultured planctomycetes class OM190 was highest on particles (2.3 ± 1.9, 3.5 ± 2.0, and 3.6 ± 2.1% on PE, PS, and wood, respectively). The relative abundance of this group in the seston samples of the treatment and control incubations was in the same range (3.3 ± 2.7 and 3.0 ± 3.5%, respectively). Abundance was highest on the seston *in situ* samples (5.1 ± 2.7%). In the free-living fraction, the highest abundance was measured in the control incubations (0.7 ± 0.4%). Among the stations, the abundance of class OM190 was highest at station MP16 (6.4 ± 1.3% on PE and 7.0 ± 2.3% on PS) and lowest at station TF0046 (0.3 ± 0.1% on PE and 1.3 ± 0.3% on PS; Figure 3).

Additional classes with relative abundances of 5–45% were either less frequent on PE, PS, and wood or showed no differences in abundance between sample types. The former included *Verrucomicrobiae*, *Planctomycetacia*, *Oxyphotobacteria*, *Bacteroidia*, and *Actinobacteria* and the latter *Phycisphaerae* and *Deltaproteobacteria* (Figure 3). Other classes were also present but their contribution to the bacterial assemblages was minor (<3%). Within this group were representatives of obligate anaerobes (such as *Kiritimatiellae*, *Anaerolineae*, and *Clostridia*), which were mainly found in the biofilms on seston (Figure 3).

### Biofilm Core OTUs and Discriminant OTUs for Plastics

**FIGURE 4:**
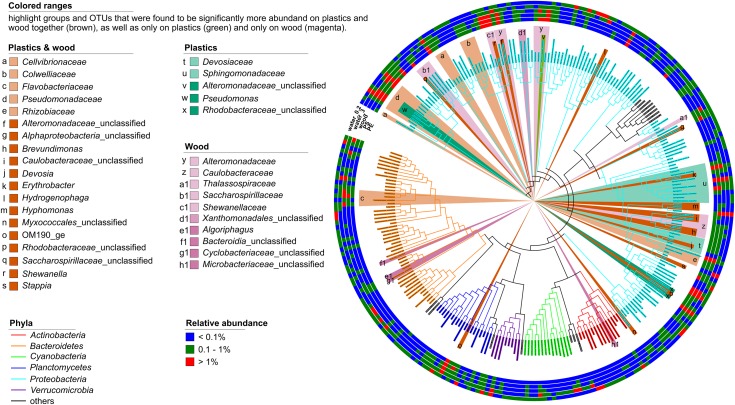
Phylogenetic tree of all bacterial OTUs with a relative abundance >0.1% in at least one sample type after 7 days of incubation. Outer rings show the relative abundances of OTUs in the PE, PS, and wood biofilms, and on seston (≥3 μm, water_3) and in the free-living fraction (3–0.22 μm, water_0.2) for both the treatment and the control incubations combined. The branch color depicts the phylogenetic affiliation of the OTUs; the background color-ranges highlight the phylogenetic groups or OTUs that differentiated the assemblages on PE, PS, and wood (brown) vs. those on seston and in the free-living fraction, and those on plastics (green) and wood (magenta) alone. *Proteobacteria* were discriminant for plastics but, for clarity, are not highlighted.

*Proteobacteria* were significantly more abundant on the plastics but were exclusively represented by *Alpha*- and *Gammaproteobacteria*. Within these two classes, the families *Devosiaceae* and *Sphingomonadaceae* were significantly more abundant [linear discriminant analysis (LDA) scores 3.8 and 4.6, respectively, *p* < 0.001]. The *Devosiaceae* were represented by OTUs of the genera *Devosia* (2 OTUs) and *Pelagibacterium* (1 OTU). Among the *Sphingomonadaceae*, 5 OTUs could not be further classified, but 2 OTUs belonged to the genus *Sphingobium*, and 1 OTU each to the genera *Erythrobacter* and *Sphingorhabdus*. Three OTUs from the genus *Pseudomonas* (LDA scores 4.2 and 3.2, *p* < 0.001), one unclassified OTU representing *Alteromonadaceae* (LDA score 4.0), and another representing *Rhodobacteraceae* (LDA scores 4.2 and 3.2, *p* < 0.001) were also discriminant for the plastics (Figure 4). The presence of some of the discriminant groups on the plastics correlated with environmental parameters. Thus, the relative abundance of *Sphingomonadaceae* correlated negatively and that of *Pseudomonas* positively with salinity (ρ = -0.83 and ρ = 0.85, respectively). Members of the *Devosiaceae* correlated negatively with ${\text{PO}}_{4}^{3-}$ concentrations (ρ = −0.79). Wood and plastics shared a core assemblage of 19 phylogenetic groups compared to the 5 differential phylogenetic groups unique to the plastic-associated assemblages (Figure 4). *Alpha-* and *Gammaproteobacteria* were the phylogenetic groups that contributed most to the differential features of the core assemblage of the combined plastics and wood samples (9 and 7 members, respectively) whereas *Deltaproteobacteria*, the uncultured planctomycetes class OM190 and *Bacteroidia* contributed one member each (Figure 4).

### *Vibrio* spp. Relative Abundances *in situ* and After 7 Days of Incubation

The relative abundances of *Vibrio* spp. were higher on PE, PS, and wood than in the *in situ* samples, but differences were also detected depending on the geographic location. In all samples, the *Vibrio* population was consistently dominated by 1 OTU. From the 13 OTUs classified as *Vibrio*, 1 OTU (OTU 137) comprised 99.6% of all *Vibrio* spp. reads. This OTU was not identical to the *V. vulnificus* OTU used as the sequencing positive control.

**FIGURE 5:**
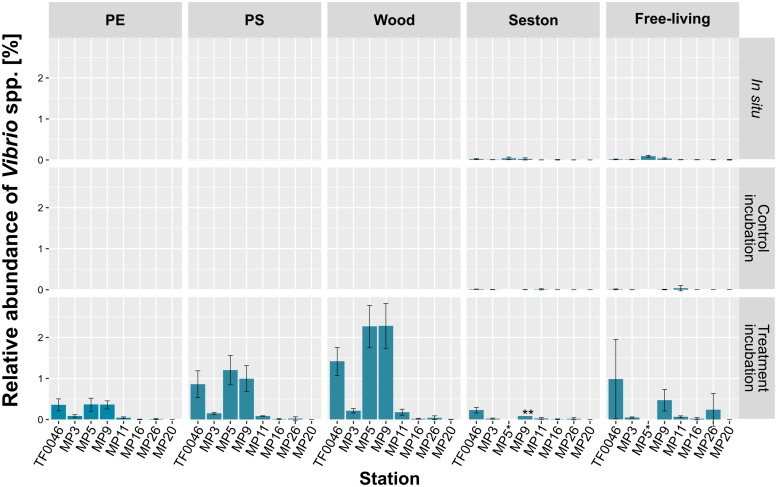
Mean relative abundances of each *Vibrio* OTU on seston (≥3 μm) and in the free-living fraction (3–0.22 μm) at the different stations at *t*_0_ (*in situ*) and after 7 days of incubation on PE, PS, wood, and seston and in the free-living fraction. Data for both the treatment and the control incubations are shown. Bars indicate the standard deviation of the most abundant *Vibrio* OTU. ^*^For station MP5, incubation water samples were not available; ^∗∗^only two replicates.

In general, the relative abundances of *Vibrio* spp. were significantly higher on samples from the treatment than from the control incubations or compared to the *in situ* samples (*p* < 0.001, Figure 5). Within the samples of the treatment incubations, relative abundances were higher on PE (0.2 ± 0.2%) and PS (0.4 ± 0.5%) than on seston (0.1 ± 0.1%), but were twice as high on wood (0.8 ± 1.0%). The concentrations of *Vibrio* spp. in the free-living fraction of the treatment incubations were in the range of those of the PE and PS samples (0.3 ± 0.5%) but were significantly higher than in either the free-living fraction of the control incubations or the *in situ* free-living fraction (*p* < 0.001 and *p* = 0.02, respectively, Figure 5 and Supplementary Table 3).

There was a clear difference in the relative abundance of *Vibrio* spp. between the different stations (79 out of 120 pair-wise comparisons were significant at *p* < 0.001–0.048; Supplementary Table 3). Thus, significantly higher abundances were determined at stations MP5 (0.4 ± 0.2% on PE, 1.2 ± 0.4% on PS, and 2.3 ± 0.5% on wood), MP9 (0.4 ± 0.1, 1.0 ± 0.3, and 2.3 ± 0.5%, respectively), and TF0046 (0.4 ± 0.1, 0.9 ± 0.3, and 1.4 ± 0.3%, respectively; *p* < 0.001–0.043; Supplementary Table 3). At the other stations, the mean relative abundances across all sample types were ≤0.1% (Figure 5). To distinguish between the effects of sample type and stations on the relative abundances, Kruskal–Wallis tests and Conover–Iman pair-wise comparisons were conducted between sample types at each station. The relative abundances of *Vibrio* spp. differed significantly between sample types at stations TF0046, MP3, MP5, MP9, and MP11 (*p* = 0.01–0.04; Supplementary Table 3). In the Spearman correlation based on environmental parameters, only the wood samples were chosen, since they had the highest numbers of *Vibrio* reads. In these samples, the only positive correlation of *Vibrio* spp. was with salinity (ρ = 0.76).

### Factors Influencing the Bacterial Assemblages

**FIGURE 6:**
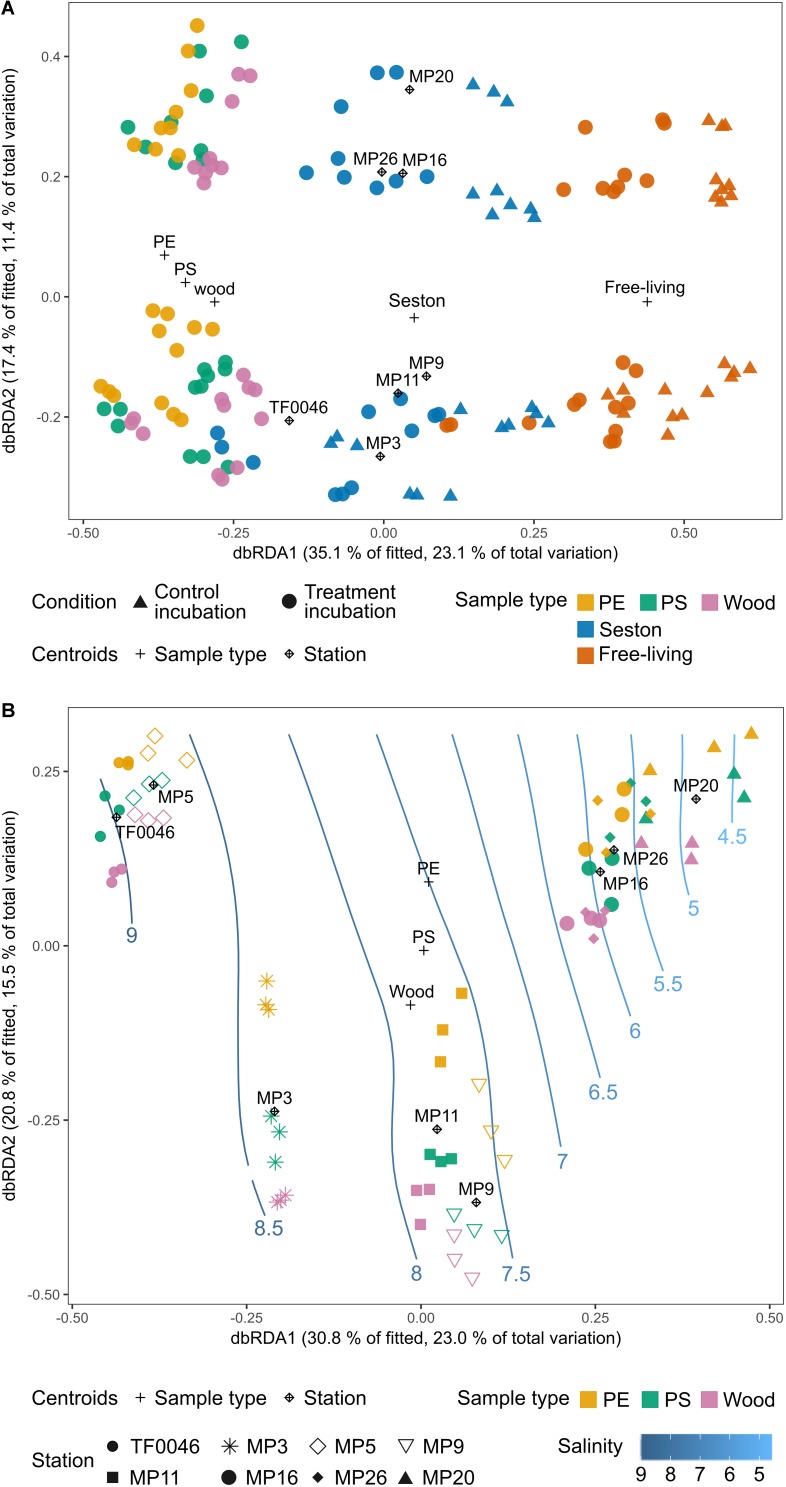
Distance-based redundancy analysis (dbRDA) ordination plots (type I scaling) based on the Bray–Curtis dissimilarities of the square-root transformed bacterial OTU read counts of **(A)** the incubated test particles (PE, PS, wood), seston (≥3 μm), and in the free-living fraction (3–0.22 μm) for both the treatment (filled circles) and control (filled triangles) incubations after 7 days and **(B)** the incubated test particles (PE, PS, wood) after 7 days. The plus sign and rhombus-shaped symbols depict the centroids of the constraining factors (sample type and station) used in the dbRDA model. Smooth response surfaces for salinity were fitted using penalized splines with the function “ordisurf” from the vegan package. For station MP5, no water samples are available and the data were therefore omitted from part **A**.

The sample type was the most important factor driving bacterial assemblage differentiation, with a clear distinction between assemblages on PE, PS, and wood versus on seston and in the free-living fraction. There was also a trend separating the PE and wood assemblages; however, when the artificially introduced substrates were investigated alone, spatial factors were dominant in shaping the biofilm assemblages. Overall, more of the variation in the complete dataset after 7 days of incubation was explained by the sample type than by the spatial factor (Figure 6A). In the dbRDA plot, the different sample types formed three clusters distributed along the first axis. Centroids of the factor “sample type” strongly correlated with the first dbRDA axis (ρ = 0.86). The assemblages on PE, PS, and wood were always significantly different from those on seston and in the free-living fractions. This was the case across all stations, independent of whether the setson and free-living samples were those of the treatment or control incubations (*p* = 0.011–0.043, Supplementary Table 4).

However, the bacterial assemblages also differed significantly between stations (*p* = 0.001) and were clearly distributed along the second dbRDA axis according to station, forming two major clusters that separated stations TF0046, MP3, MP9, and MP11 from stations MP16, MP20, and MP26 (Figure 6A). Centroids of the spatial factor “station” strongly correlated with the second dbRDA axis (ρ = 0.83). Together, the first two axes explained 52.5% of the fitted variation while the full dbRDA model explained 63.1% of the variation in the bacterial assemblages between the PE, PS, wood, seston, and free-living fraction samples. Both were significant contributors to explaining the variation (*p* = 0.001) while the variation was significantly partitioned by the dbRDA axis (*p* = 0.001).

Tests of the homogeneity of the multivariate dispersions within groups did not yield significant results for the factor “station” (*p* = 0.54), whereas in some cases significant results were obtained in the global test of the factor “sample type” (*p* = 0.001). However, when the sample types were tested within the subset of each station, none of the pair-wise comparison produced a significant result (*p* = 0.22–1, Supplementary Table 4).

When only the assemblages on PE, PS, and wood were compared, more of the variation between the bacterial assemblages on PE, PS, and wood after 7 days of incubation was explained by the spatial factor than by sample type. “Station” was a significant factor (*p* = 0.001, Figure 6B) and its centroids strongly correlated with the first dbRDA axis (ρ = 0.91), which explained 30.8% of the fitted variation. The samples formed four clusters along this axis: stations TF0046 and MP5 clustered together, as did stations MP9 and MP11; station MP3 formed a separate cluster between those two clusters and stations MP16, MP20, and MP26 formed a fourth, distinct cluster (Figure 6B). These clusters were significantly different, as shown in pair-wise PERMANOVAs (*p* = 0.001). The factor “sample type” was still a significant contributor to explaining the variation (*p* = 0.001) but it was not one of the main factors and its centroids did not correlate with the first or second dbRDA axis (*ρ* = 0.05 and 0.21, respectively). The first two axes explained 51.6% of the fitted variation and the full model 71.1% of the total variation. Both the full model and the axes significantly captured the variation within the bacterial assemblages on PE, PS, and wood after 7 days of incubation (*p* = 0.001, respectively). The assemblages on PE differed significantly from those on wood at all stations (*p* = 0.02–0.042, Supplementary Table 4), except at stations MP20 (*p* = 0.075) and MP16 (*p* = 0.054). The assemblages on PS differed significantly from those on wood only at station MP26 (*p* = 0.039) whereas those on PE and PS differed significantly only at station MP3 (*p* = 0.035, Figure 6B). The fitted response surfaces for the environmental parameters were also significant (*p* < 0.001), but salinity explained most of the variation (96.7%) and was also gradually arranged along the first dbRDA axis. It was therefore chosen for display in the dbRDA plot (Figure 6B and Supplementary Figure 4).

## Discussion

In this study, young biofilms on PE and PS as well as on wood, as a natural polymer, were investigated with respect to the influence of environmental factors and different surfaces. The results were then compared to those from bacteria inhabiting seston and in the free-living water fraction. Most of the variation in bacterial assemblages could be explained by the sample type but environmental factors were dominant in the structuring of biofilm assemblages on PE, PS, and wood. The relative abundances of *Vibrio* spp. were compared, on the different materials, both in the different incubations and vs. seston-attached and free-living bacteria *in situ*. The results showed that while *Vibrio* numbers were elevated on PE and PS, they were highest on wood.

### Microplastics Comprise a Newly Available Habitat for Biofilm-Forming Bacteria in Aquatic Ecosystems

Our results confirm that microplastics comprise a novel habitat in the Baltic Sea for surface-attached bacteria, as already shown in the Mediterranean Sea and in previous studies of the Baltic Sea (Dussud et al., 2018b; Oberbeckmann et al., 2018; Ogonowski et al., 2018). Despite a coastline of ∼2000 km and waters of different salinities and anthropogenic inputs, in our study of the Baltic Sea the sample type was still the major factor explaining the differences between the bacterial assemblages on PE, PS, and wood vs. those on seston and in the free-living fraction. However, it should be noted that the biofilms on PE, PS, and wood were only 7 days old, whereas neither the age of the seston nor its colonization history could be determined. Chao1 richness and the number of unique OTUs were lowest in the PE and PS samples, which suggests differences in the succession stages of the introduced particles vs. of seston at *t*_7_. However, the Chao1 richness determined for wood did not differ significantly from that determined for seston. It is therefore unlikely that the dissimilarity between the bacterial assemblages on PE, PS, and wood vs. either on seston or in the free-living fraction can solely be attributed to differences in succession stages. Instead, an effect of substrate type on the developing assemblages is more likely, as also shown in other *in situ* studies (Dussud et al., 2018b; Oberbeckmann et al., 2018). Differences in the assemblages present on inert surfaces and in bacteria colonizing natural aggregates of biogenic origin have also been reported for stream ecosystems (Niederdorfer et al., 2016). Although in this study the generally lower Chao1 richness within the incubation tanks might have resulted from a bottle effect arising from the incubation, comparisons among the incubations were still valid. There were also clear differences in the relative abundances of specific phylogenetic groups. *Gammaproteobacteria* was the most dominant group after the 7 days of incubation and was more abundant on PE, PS, and wood than on the *t*_7_ seston samples and in the *t*_7_ free-living fraction. Both copiotrophic species and species identified in batch cultures as primary responders are found within the *Gammaproteobacteria* class (Eilers et al., 2000). There was a general increase of *Gammaproteobacteria* from the *in situ* samples to samples obtained from the incubation tanks at day 7, which can in part be attributed to the incubation conditions. However, other studies of aquatic biofilms also showed that *Gammaproteobacteria* are usually among the early colonizers of inert surfaces (Dang et al., 2008; Lee et al., 2008; Li et al., 2014; Lawes et al., 2016; De Tender et al., 2017; Dussud et al., 2018a), which according to this study includes those present in the Baltic Sea. As copiotrophs, *Gammaproteobacteria* may be able to quickly respond to the enhanced availability of the organic substances, such as proteins and polysaccharides, that adsorb to immersed surfaces.

The *Alphaproteobacteria* comprised the second most abundant group on PE, PS, and wood after 7 days of incubation. Members of the *Alphaproteobacteria* are also consistently identified as primary colonizers of surfaces in aquatic systems, especially the marine *Roseobacter* clade within the family *Rhodobacteraceae* (Dang et al., 2008). Unclassified members of the *Rhodobacteraceae* were abundant on PE and PS, with ∼60% of the sequences affiliated with genera within the *Roseobacter* clade (Supplementary Figure 5), indicating that taxa usually found in marine biofilms contribute to the young biofilm assemblages. Studies on the initial colonization of surfaces immersed in marine waters have shown that during the first 24 h of biofilm formation *Gammaproteobacteria* were the first group to colonize glass, acrylic glass, steel, and polyvinylchloride; thereafter, the surfaces were rapidly dominated by *Alphaproteobacteria* (Dang et al., 2008; Lee et al., 2008). However, studies explicitly investigating biofilm formation on artificial polymers (PE and acrylic glass) found that *Gammaproteobacteria* can dominate the assemblages during the first 7 days of incubation (Li et al., 2014; Lawes et al., 2016; De Tender et al., 2017; Dussud et al., 2018a), which may hint toward a general trend of preferential biofilm formation by *Gammaproteobacteria* on artificial polymers. *Bacteroidia*, as the third most abundant class on PE, PS, and wood, are also well known biofilm-forming bacteria occurring within marine and brackish systems (DeLong et al., 1993; Elifantz et al., 2013). Whereas they are usually the first to respond to particulate organic matter inputs such as those deriving from phytoplankton blooms (Teeling et al., 2012), on inert surfaces *Bacteroidia* seem to be late colonizers (De Tender et al., 2015, 2017), a strategy that may allow them to take advantage of the release of organic compounds by primary colonizing organisms. This would explain the relatively low abundance of *Bacteroidia* in the young biofilms on PE and PS vs. on seston after only 7 days of incubation.

These results confirm that biofilms on PE, PS, and wood form a habitat distinct from that of seston. However, differences between the bacterial assemblages on these three substrates were difficult to determine, despite the significantly higher Chao1 richness of the assemblages on wood. The latter observation can be attributed to the greater surface heterogeneity of wood, with its pits and cracks providing a larger number of possible microhabitats than available on the more homogeneous surface of plastics (Horner-Devine et al., 2004). Also, a pronounced phylogenetic overlap was determined between taxa discriminant for plastics only vs. plastics and wood combined. The family *Devosiaceae* was a discriminant group for plastics, but the genus *Devosia*, within the *Devosiaceae*, was also a discriminant group for plastics and wood combined. Likewise, the genus *Pseudomonas* was a discriminant taxon for plastics alone, but the family *Pseudomonadaceae* was a discriminant group for plastics and wood. Very few features were discriminant for plastics only compared to plastics and wood. Thus, in this study, the majority of the colonizing organisms in the young biofilms that formed on PE and PS were general biofilm-forming taxa rather than surface-specific specialists.

Nonetheless, the families *Sphingomonadaceae* and *Devosiaceae*, the genus *Pseudomonas*, and unclassified *Rhodobacteraceae* and *Alteromonadaceae* were significantly more abundant on the plastics at *t*_7_. Many members of these groups are able to form biofilms (Dang and Lovell, 2002; Stolz, 2009; López-Pérez and Rodriguez-Valera, 2014; Masák et al., 2014). For example, *Sphingomonadaceae*, such as *Erythrobacter*, *Sphingopyxis*, and *Sphingomonas*, have consistently been found in biofilms on microplastics, thus demonstrating that our results adequately reflect *in situ* conditions (Zettler et al., 2013; Hoellein et al., 2014; Jiang et al., 2018; Oberbeckmann et al., 2018; Ogonowski et al., 2018). Moreover, these organisms may represent core species of the plastic-associated microbiome. Many *Sphingomonadaceae*, including members of the genera *Erythrobacter* and *Sphingobium*, which in this study were significantly more abundant on plastics, as well as members of the genera *Pseudomonas* and *Devosia* have been described as putative hydrocarbon degraders and have repeatedly been isolated from environments contaminated with petroleum-derived hydrocarbons (Onaca et al., 2007; Kumar et al., 2008; Stolz, 2009). They are also abundant in the biofilms that form on other organic surfaces in aquatic systems, such as brown and green algae (Staufenberger et al., 2008; Burke et al., 2011; Lachnit et al., 2011). The consistent detection of these bacteria on natural and petroleum-derived polymers has been linked to the potential degradation of marine microplastics by the respective species (Zettler et al., 2013; Dussud et al., 2018a; Ogonowski et al., 2018). Our results show that these organisms are also members of the young biofilms that develop on microplastics in the Baltic Sea. However, nothing is known whether these organisms are able to degrade the carbon-backbone of the polymers. A first metagenome study of the microplastic-associated assemblages revealed an overrepresentation of genes involved in xenobiotic degradation processes (Bryant et al., 2016), but it may also be the case that the bacteria take advantage of the volatile compounds released from the plastics even after 2 weeks, such as monomers and additives (Romera-Castillo et al., 2018; Klaeger et al., 2019), or make use of the organic pollutants that sorb to the surface of the polymers (Mato et al., 2001). These scenarios warrant further investigation.

Despite the relatively small differences between the assemblages on PE and PS vs. on wood, our study shows that plastics, as newly introduced hard substrates, are colonized by biofilm consortia that differ from those found on natural seston. Given the current quantity of plastic debris in the ocean and the predicted increase thereof (Thompson R.C. et al., 2004), the difference between plastic and natural surfaces might be negligible, with the large quantity of hard substrates newly introduced into a system that is otherwise devoid of such habitats being of much greater ecological relevance. The impact this development can have on aquatic ecosystems and its functioning needs to be acknowledged and should be carefully investigated.

### The Vector Potential of Microplastics for *Vibrio* Depends on the Life History of the Particle

The relative abundance of *Vibrio* spp. was determined to facilitate comparisons across both sampling types measured in different units and different studies. Accordingly, the relative abundances of *Vibrio* spp. on PE and PS in this study were lower than those reported by Zettler et al. (2013) (24% on one sample) and Frère et al. (2018); (up to 19%). However, they were higher than the *in situ* amounts of *Vibrio* spp. on seston and in the free-living fraction collected during the study cruise. The abundances on PE, PS, and wood were also higher than those reported for free-living *Vibrio* occurring in the vicinity of the Stockholm Archipelago, where the maximum was 0.002% (calculated from data in Eiler and Bertilsson, 2006; Eiler et al., 2006). Thus, the *in situ* abundances in the free-living fraction (max. 0.09%) measured in our study were comparable to those of earlier studies and consistent with the increased abundances found on PE and PS. However, relative abundances were highest on wood (max. 2.3%), which indicated that the detected *Vibrio* OTU represented a biofilm generalist, a conclusion well in line with the findings of Oberbeckmann et al. (2018).

The relative abundances of *Vibrio* spp. on PE (max. 0.4%) and PS (max. 1.2%) in this study were higher than in most of the reported occurrences described in other studies investigating floating plastic debris in the ocean. In those studies, *Vibrio* spp. abundances ranged between 0.0032 and 0.6% (Schmidt et al., 2014; Debroas et al., 2017; Dussud et al., 2018b; Jiang et al., 2018; Oberbeckmann et al., 2018). The use of PCR-amplified amplicon sequencing in this study may have introduced a PCR-related bias (Polz and Cavanaugh, 1998). However, the *Vibrio* numbers detected are comparable to those previously obtained in a similar experimental set-up in which abundances were determined using a combination of amplicon sequencing and quantitative PCR (Oberbeckmann et al., 2018) and to the *Vibrio* abundances measured in the Baltic Sea using a quantitative competitive PCR approach (Eiler and Bertilsson, 2006), such that a severe over- or underestimation of *Vibrio* quantities in this study was unlikely. Also, the *Vibrio* abundances in the treatment incubation were significantly higher than in the control incubation after 7 days, which clearly showed that the increase in *Vibrio* spp. was not an incubation artifact.

The genus *Vibrio* is considered an r-strategist. While it is usually found in low numbers (<0.1%) throughout the world (Eilers et al., 2000; Thompson J.R. et al., 2004), it quickly responds to nutrient inputs (Eilers et al., 2000; Takemura et al., 2014) to reach high abundances, a reaction attributed to high growth rates and high rRNA copy numbers (Heidelberg et al., 2000; Gilbert et al., 2012; Westrich et al., 2016). This “feast or famine” strategy might explain the elevated relative abundances detected on the PE, PS, and wood particles in this study after only 7 days of incubation. Thus, the identified *Vibrio* OTU may have been among the organisms able to take early advantage of the new habitats as well as the nutrients in the conditioning film. Indeed, a study investigating early succession on chitin particles showed that vibrios were among the very early colonizers (Datta et al., 2016).

*Vibrio* numbers were elevated only in the incubations with water from stations TF0046, MP5, and MP9, i.e., from Mecklenburg Bay to the Bay of Gdansk, where the salinity range is 7.7–9 PSU. The lower *Vibrio* abundances in the incubations with water from the other stations indicated that the detected *Vibrio* OTU was present along the southeastern Baltic Sea coast, but that its optimal growth occurred at salinities >7 PSU. Hood and Winter (1997) found that the attachment of different *V. cholerae* strains to surfaces occurred primarily at NaCl concentrations of 1%. The attachment of *V. cholerae* and other *Vibrio* species was also shown to be impaired in the presence of low Ca^2+^ concentrations (Kierek and Watnick, 2003a, b), characteristic of freshwater and waters of lower salinity (Schubert et al., 2017). The significantly lower abundances of *Vibrio* spp. on PE, PS, and wood from the station MP3 incubations, in which the mean salinity was 8.2, suggested that additional factors play a role in the contribution of *Vibrio* spp. to biofilm formation. Moreover, station MP3 had the highest Chao1 richness, such that other primary colonizers may have prevented the growth of *Vibrio* sp. by outcompeting these bacteria (Rendueles and Ghigo, 2015). Our findings could account for the sporadically high abundances of *Vibrio* on the microplastics sampled *in situ* but are otherwise inconsistently detected on them. Firstly, *Vibrio* can be regarded as member of young biofilms and a putative primary colonizer of solid surfaces and would likely be absent from older particles. Secondly, environmental conditions, e.g., nutrient availability or the lack of specific salts, may have been suboptimal for *Vibrio* biofilm formation in general. Thus, the detection of elevated *Vibrio* abundances may be indicative of relatively newly colonized particles and therefore of their possible sources. Investigations of the succession of biofilm assemblages on microplastics are needed to fully assess the temporal dynamics of *Vibrio* spp. as an early colonizer. Such studies must also take into account the “life history” of the microplastic particles to obtain a holistic risk assessment.

Chubarenko and Stepanova (2017) proposed a scheme for microplastic transport in the Baltic Sea and hypothesized that particles undergo several beaching and immersion events, which could lead to repeated cycles of colonization, before the particles sink or are otherwise removed. This scenario suggests the importance of investigating not only the spatial scale but also the temporal dynamics of biofilm formation (De Tender et al., 2017). Of note, after 7 days the relative abundances of *Vibrio* spp. were higher in the free-living fraction in water from the treatment incubation than in the control incubation. An ability of particles to affect other compartments of the aquatic system was previously demonstrated in a study showing that a close relative of the gammaproteobacterium *Amphritea atlantica* was enriched on PS and in the respective incubation water (Kesy et al., 2016). Although this effect might be overestimated in a closed system such as an incubation tank, it still shows the potential of microplastics, including their potential leachates, to alter the assemblages in their surroundings. Accordingly, not only the changes that plastic particles and their biofilms bring to aquatic ecosystems usually void of hard substrates, but also the effect of these newly introduced substrates on the free-living bacterial assemblage must be taken into account. This is of particular importance in areas with high microplastic concentrations, such as in East Asian seas (Isobe et al., 2015).

Microplastics might not be the sole vectors for potential pathogens, as higher abundance of *Vibrio* was detected on wood. Nonetheless, with the increasing burden of microplastics in the ocean, the microplastic load may become an important dispersal vector.

### Biofilm Differentiation on Microplastics Differs According to the Sampling Location, but Nutrient Limitation May Select for Surface Specificity

Oberbeckmann et al. (2014, 2016) found that location and season were prominent drivers of the biofilms that developed on PET after 6 weeks of incubation at different stations in the North Sea. Amaral-Zettler et al. (2015) demonstrated that plastic-associated biofilms sampled in the Atlantic and Pacific oceans showed biogeographic patterns that separated the assemblages found in these systems. However, other studies found no differences in the plastic-associated biofilms from different geographic locations. Dussud et al. (2018b) sampled microplastics in the western Mediterranean Basin and were unable to differentiate among the bacterial assemblages based on the sampling site. Likewise, Bryant et al. (2016) found no evidence of spatial differences along a ∼2000-km transect across the Pacific Ocean. By contrast, in our study the sampling location was the most important factor structuring the plastic- and wood-associated bacterial assemblages. This was evident from the dbRDA based on the OTU level as well as the relative abundances of higher-order phylogenetic groups. The prominent geographic influence observed in this study, in contrast to the findings of Dussud et al. (2018b) and Bryant et al. (2016) in probably more homogeneous habitats, was most likely due to the environmental heterogeneity of the Baltic Sea. Thus, *Gammaproteobacteria* were more abundant on PE, PS, and wood particles exposed to the higher salinity western stations TF0046–MP5 and less abundant at the lower salinity eastern stations MP9–MP26. The relative abundances of *Alphaproteobacteria* increased from the western toward the eastern stations (MP9–MP26) after 7 days of incubation.

We argue that salinity, and not other nutrients, was the main driver of the differentiation of the PE-, PS-, and wood-associated assemblages because salinity is the major factor differentiating bacterioplankton assemblages globally (Lozupone and Knight, 2007), including in the Baltic Sea, where bacterial assemblages were previously shown to be influenced by salinity rather than by inorganic nutrient concentrations (Herlemann et al., 2011, 2016; Rieck et al., 2015). Additionally, the three distinct clusters of the assemblages apparent from the dbRDA did not accord with the clustering of the stations when environmental parameters were considered, assuming the equal importance of each one (Supplementary Figure 6). However, a role for other factors in bacterial assemblage differentiation was suggested by the bacterial assemblages from station MP3 (mean salinity 8.2 PSU), which clustered between the tightly clustered samples from the higher saline stations TF0046 and MP5 (9–8.5 PSU) and the cluster derived from the intermediate saline stations MP9 and MP11 (7.7 PSU).

Robust conclusions regarding the factors influencing biofilm formation require investigations performed under controlled conditions (Ogonowski et al., 2018). Thus, despite the challenges posed by extrapolating the results obtained in incubation experiments to natural systems, our interpretation can be considered as valid, since all incubations were subjected to the same environmental pressure. Although *in situ* incubations are closer to natural systems, those performed along a 2000-km transect do not allow the exclusion of factors such as differences in hydrodynamics, solar radiation, or temperature, which would make any interpretation of the results even more challenging.

Of note, we were able to differentiate early biofilms even along a relatively moderate salinity gradient (4.5–9 PSU) almost exclusively within the mesohaline range (Anonymous, 1958). Differences in biofilm assemblages likely reflect already-existing differences in the respective source community, indicating the importance of the inoculum on the resulting biofilm assemblage (Crump et al., 2012; Ruiz et al., 2014). However, even when the detected phylogenetic groups were present in equal abundances in the source community at *t*_0_, differences in their relative abundances on PE, PS, and wood emerged after 7 days of incubation (e.g., *Alphaproteobacteria*), which suggests the contribution of additional factors to the community composition of young biofilms. Studies on the attachment behavior of bacterial isolates have shown that ionic composition and concentrations influence substrate adhesion, such that the degree of attachment of the same bacterial species on PE and PS may have been determined by the different salinities (Bakker et al., 2004; Karatan and Watnick, 2009). Adhesive and biofilm polymers of *Pseudomonas* spp. isolated from freshwaters and marine waters were previously shown to differ in their responses to electrolyte addition, resulting in reduced biofilm thickness in the freshwater isolate, but not in the marine strain (Fletcher et al., 1991).

Oberbeckmann et al. (2018) found that substrate type was more important at low nutrient concentrations and higher salinity than at high nutrient conditions and lower salinity. Inorganic nutrients are generally depleted in summer in the Baltic Sea, following the spring diatom bloom, with nitrogen being the most limiting nutrient (Schneider et al., 2017). This was also the case during the study period, in August and September 2015, except at stations MP16 and MP20 in the Gulf of Finland, which were not initially nitrogen-limited. Those stations also had the highest initial concentration of dissolved organic carbon (DOC). Notably, the biofilm assemblages on PE and wood did not differ significantly at these two stations, unlike at all other stations. This could have been due to a difference in the condition films, with the higher DOC concentrations at MP16 and MP20 masking the surface properties of the materials (Lorite et al., 2011). Alternatively, differences in surface specificity may depend on the adhesion capacity of the bacteria themselves. Previous studies have shown that the capacity to induce biofilm formation can depend on the nutritional status of the bacterial cells and that bacteria under nutrient-limitation differ in their surface attachment behaviors (Allan et al., 2002; Karatan and Watnick, 2009). In general, these findings corroborate the results of Oberbeckmann et al. (2018) and further suggest that, even at overall low inorganic nutrient concentrations, nutrient ratios could play a role in determining surface specificity. Further research on the role of the conditioning film in surface specificity vs. whether and how limitations in inorganic nutrients serve as a driver of surface-specific bacterial attachment on diverse microplastics is needed.

To date, we still do not know much about the dynamics and successional changes in microplastic-associated assemblages that occur as the particles are subjected to different environments characterized by different local communities, such as during transport by currents and winds. Studies thus far have shown that microplastic-associated biofilms are unstable after a disturbance and that the local environment acts as a selective force (Kesy et al., 2016, 2017).

## Conclusion

Even along a moderately distinct environmental gradient, the assemblages on PE, PS, and wood differed in terms of their Chao1 richness and composition from assemblages on seston and in the free-living fraction. This observation demonstrated the importance of location in determining the assemblages on these three substrates. Our study also showed that the formation of surface-specific biofilms may depend on inorganic nutrient availability and that the relative abundances of the dominant *Vibrio* OTU in the young biofilms that formed on PE, PS, and wood were linked to geographic location and correlated positively with salinity. Thus, while microplastics comprise a novel habitat for biofilm-forming bacteria, environmental factors, especially salinity, greatly influence the composition of biofilm assemblages. In contrast to other studies, we detected a higher abundance of *Vibrio* spp. on microplastics but also on wood, consistent with a role for *Vibrio* in young biofilms. Taken together, our results highlight the need to take into account spatial factors, the temporal dynamics of biofilm formation and the “life history” of the particles to assess the full importance of microplastics as a new habitat and potential vector for surface-associated bacteria in aquatic systems.

## Author Contributions

KK, SO, and ML designed the experiment and analyzed the data. KK wrote the manuscript. ML, SO, and BK provided invaluable comments and intellectual input. BK provided the laboratory equipment and measurement data. All authors read and approved the final version of the manuscript.

## Conflict of Interest Statement

The authors declare that the research was conducted in the absence of any commercial or financial relationships that could be construed as a potential conflict of interest.
