# Supplementary material for: Spatial Environmental Heterogeneity Determines Young Biofilm Assemblages on Microplastics in Baltic Sea Mesocosms
Source: Front Microbiol. 2019 Aug 9;10:1665. doi: 10.3389/fmicb.2019.01665 (PMC6696623; doi:10.3389/fmicb.2019.01665)
Supplement: Supplementary file 1 [file Data_Sheet_1.pdf]

Supplementary Material

Spatial environmental heterogeneity determines young biofilm assemblages on microplastics in Baltic Sea mesocosms

Katharina Kesy[1], Sonja Oberbeckmann[1], Bernd Kreikemeyer[2] , Matthias Labrenz[1*]

[1]Biological Oceanography, Leibniz Institute for Baltic Sea Research Warnemuende (IOW), Rostock, Germany

[2]Institute of Medical Microbiology, Virology and Hygiene, University Medical Center Rostock, Rostock, Germany

*** Correspondence:** matthias.labrenz@io-warnemuende.de

Supplementary Material & Methods

Hierarchical clustering of stations according to environmental parameters

For comparing physico-chemical parameters of the stations, hierarchical clustering based on Euclidian distance of the z-transformed data was conducted using the Ward method. The means between the t_0 and t_7 data were used as input. All calculations were done in the R program for Statistical Computation (R Core Team, 2017) using the function 'vegdist' for Euclidean distances (Oksanen et al., 2018) and 'base'-functions for transformations ('scale') and hierarchical clustering ('hclust').

Unclassified *Rhodobacteraceae* tree

To gain insight into the phylogenetic affiliations of the unclassified *Rhodobacteraceae*-OTUs, the representative sequences for these OTUs only found on the PE and the PS were retrieved using the mother commands 'get.oturep' and 'get.lineage'. The OTUs present only on the PE and the PS were then picked manually, resulting in 116 representative sequences. These were aligned using the SINA online tool (Pruesse et al., 2012). Aligned sequences were in loaded into the complete bacterial SSU tree Ref. Nr. 99 Release 132 in ARB (Ludwig et al., 2004) using ARB parsimony. Neighboring sequences as well as close type strains were then chosen and a baseline tree was constructed only with these sequences using the Neighbor Joining method with bootstrapping (1000) and Jukes-Cantor correction. The genus *Acidimicrobium* (*Actinobacteria*) was used as outgroup. The unclassified *Rhodobacteraceae* sequences were then added into this baseline tree using ARB parsimony. The tree was visualized using the iTOL online tool (Letunic and Bork, 2016).

Supplementary Figures

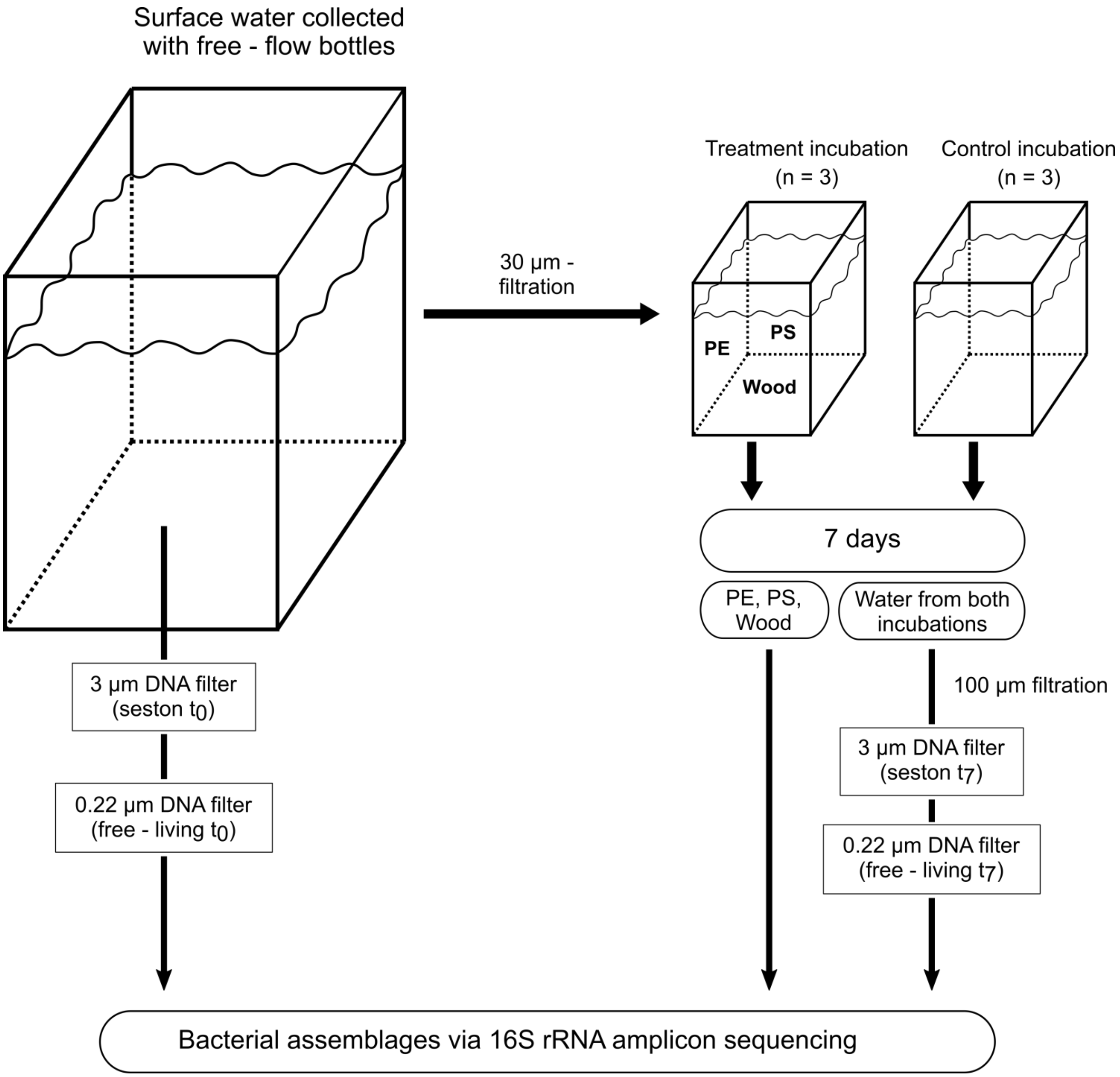

Supplementary Figure 1. Schematic overview over the experimental set-up and sampling procedure. Surface water from within the first 5 m was collected using a CTD-rosette equipped with free-flow bottles. Water from the bottles was mixed to create a homogenous starting community (represented as the bigger tank). Seston-attached bacteria of the t_0 community (*in situ*) were collected on 3 µm pore-size filter, and the free-living bacterial fraction on 0.22 µm pore-size filters (3 technical replicates). The water was then 30 µm filtered to exclude bigger grazers and distributed into incubation tanks (1.5 L). Polyethylene (PE), polystyrene (PS) and wood pellets were introduced into the treatment incubation, a control was run without the introduction of pellets (control incubation). Incubations were run for 7 days at ambient temperature (20°C) and a light/dark rhythm between 19/5 h and 18/6 h. Incubations were aerated with aquarium diffuser stones.

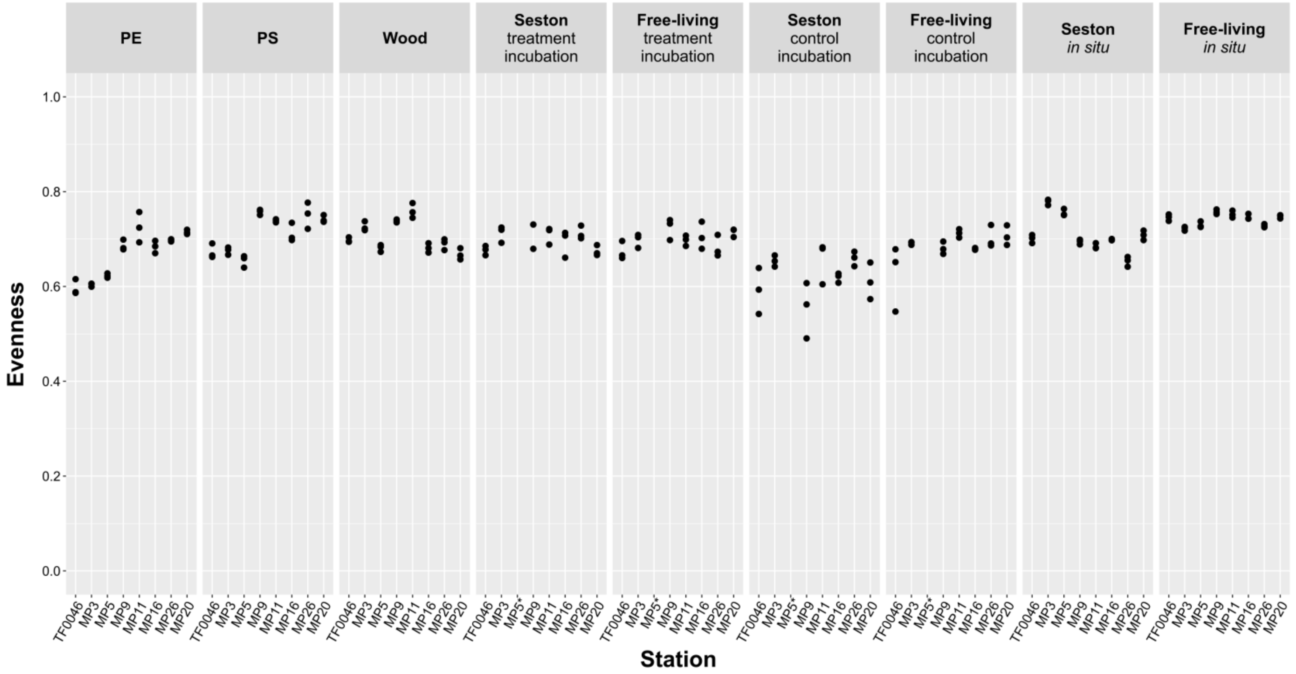

Supplementary Figure 2. Pielou's evenness based on bacterial OTUs on seston (≥ 3 µm) and in the free-living fraction (3 – 0.22 µm) of the water at different stations at t_0 (*in situ*) and after 7 days of incubation on the PE, PS and wood, and on seston and in the free-living fraction for both the treatment and control incubations. *For station MP5, incubation water samples were not available.

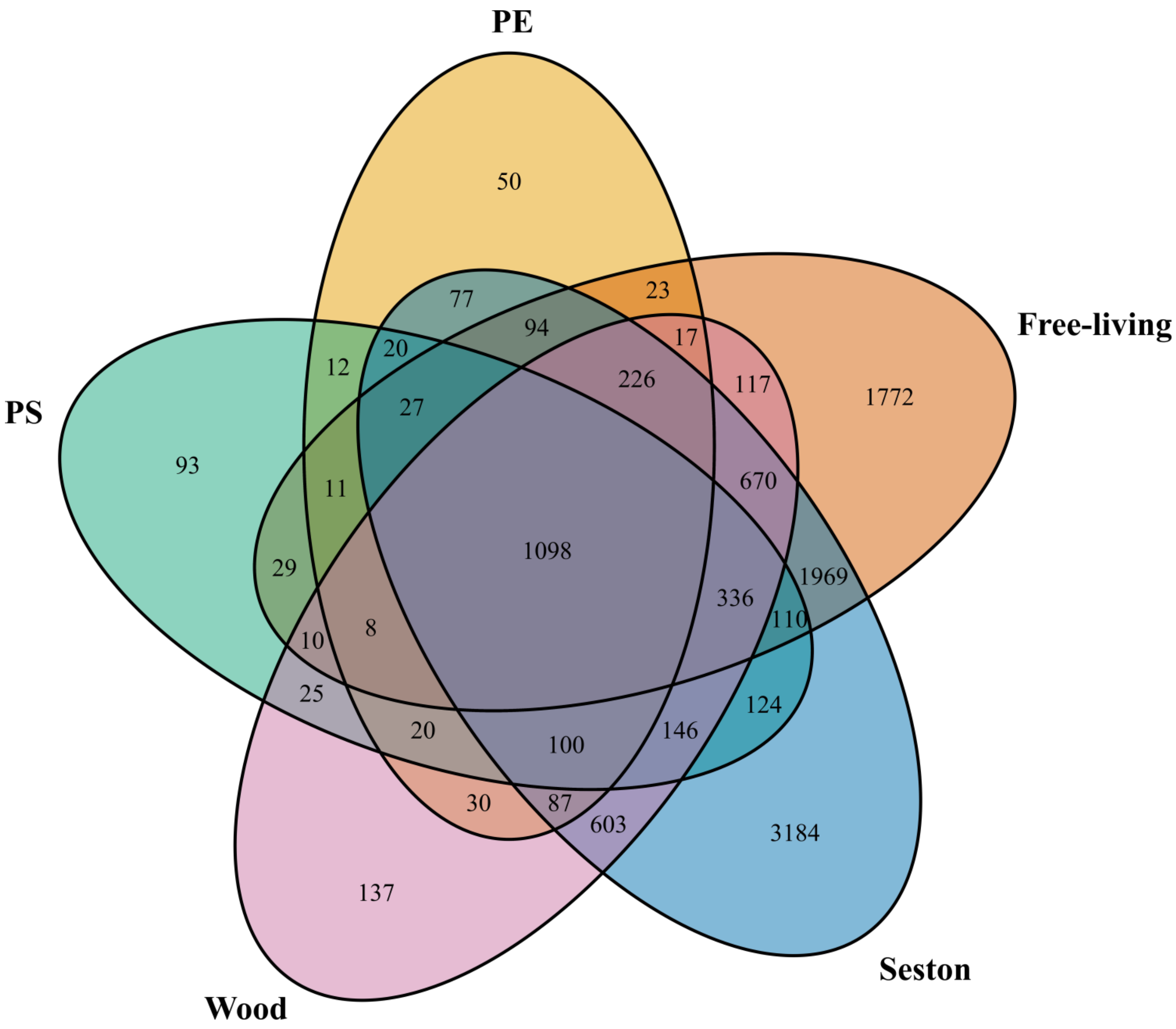

Supplementary Figure 3. Venn diagram depicting unique and shared fraction of bacterial OTUs on the PE, PS and wood after 7 days of incubation in seawater and assemblages on seston (≥ 3 µm) and in the free-living fraction (3 – 0.22 µm) of the incubation water of the treatment- and the control incubations at t_0 and t_7 combined.

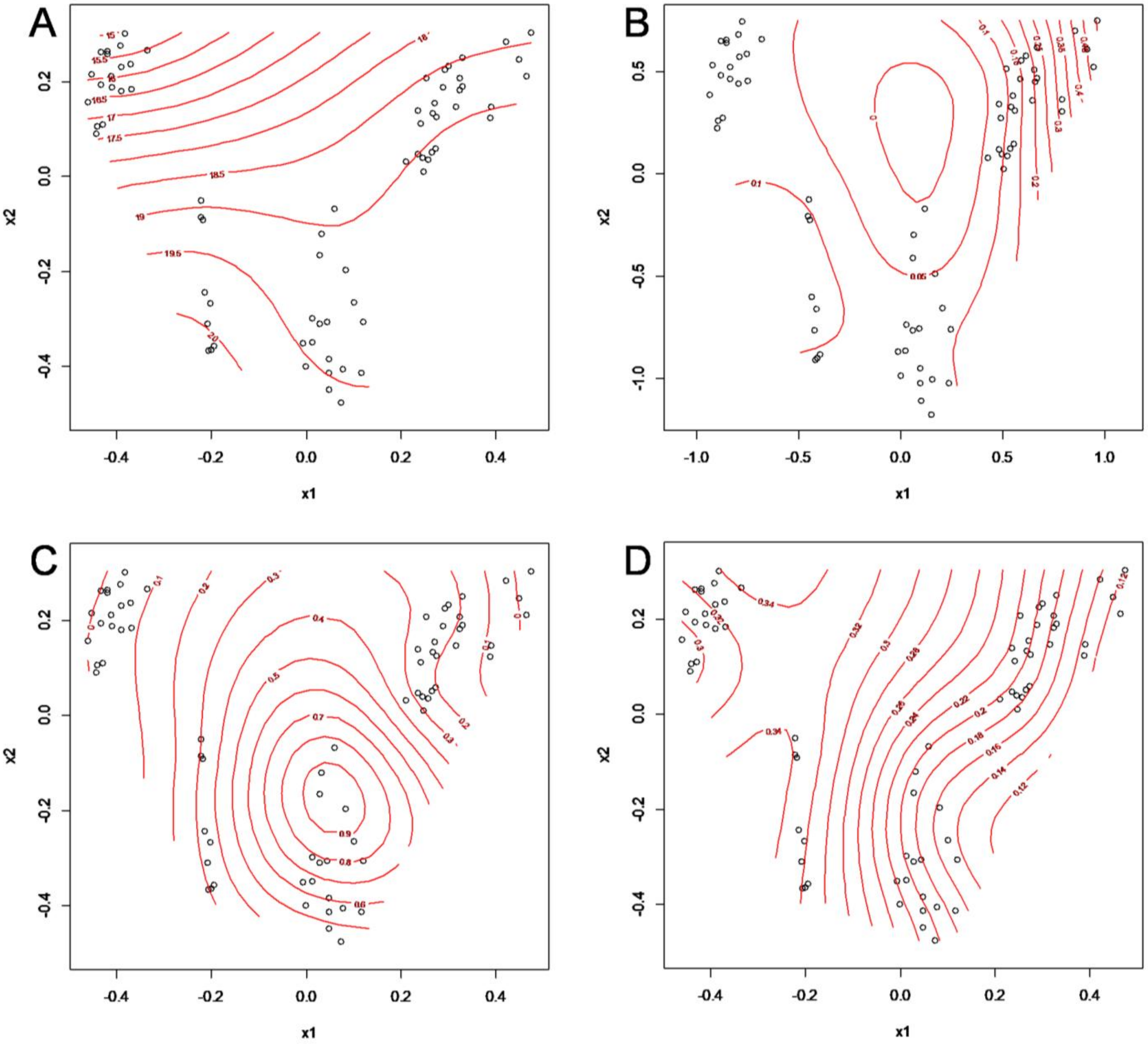

Supplementary Figure 4. Distance-based redundancy analysis (dbRDA) ordination plots (type I scaling) based on the Bray-Curtis dissimilarities of the square-root transformed bacterial OTU read counts of the incubated PE-, PS- and wood particles after 7 days. Smooth response surfaces for (**A**) temperature, (**B**) NO_2^-, (**C**) NO_3^-, and (**D**) PO_4^{3-} were fitted using penalized splines with the function ‘ordisurf’ from the vegan package.

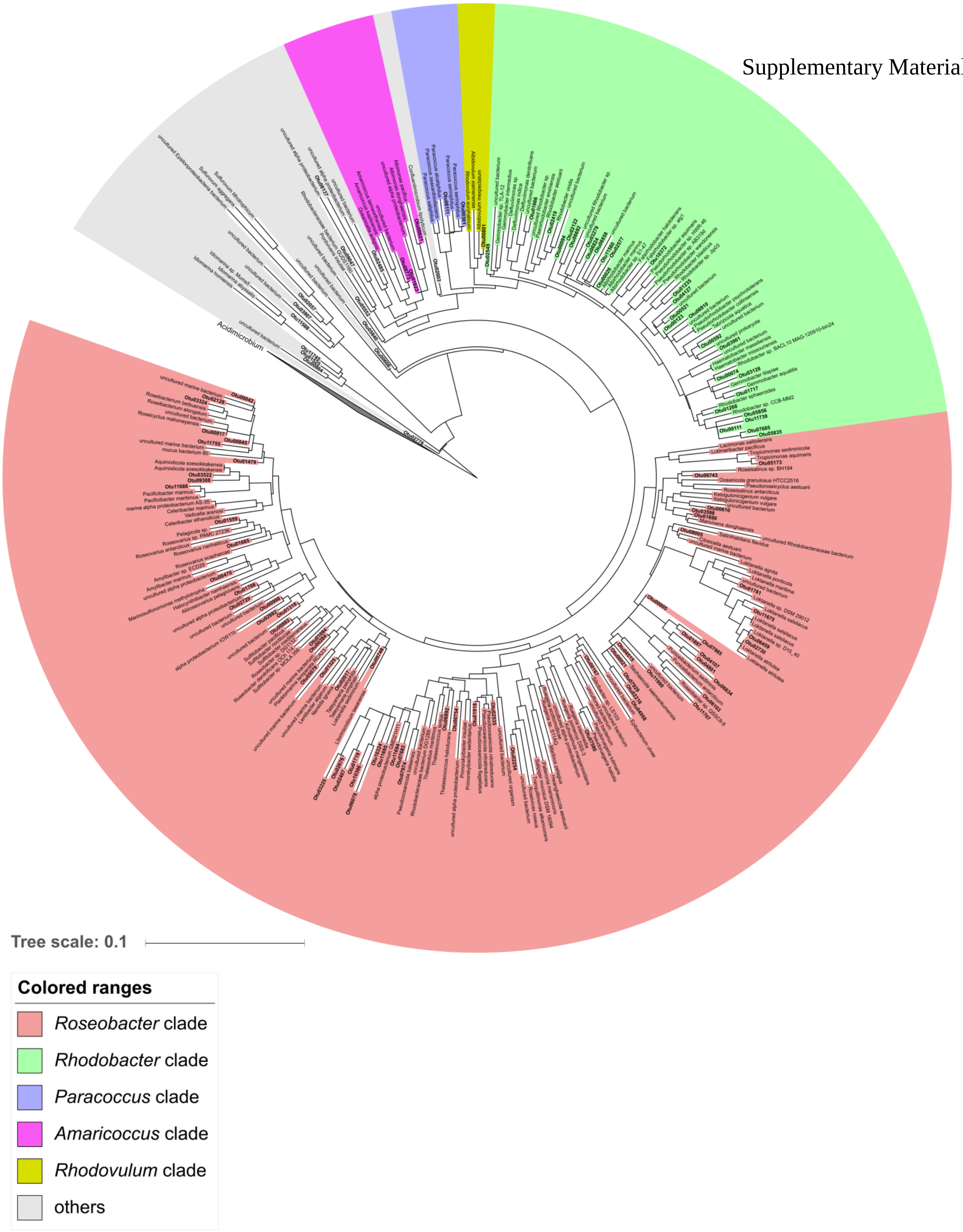

Supplementary Figure 5. Phylogenetic affiliations of unclassified *Rhodobacteraceae*-OTUs present only on the incubated PE and PS. Colored ranges highlight affiliation of sequences with distinct clades within the family *Rhodobacteraceae*. Entries highlighted in bold represent OTUs found in this study. Collapsed nodes represent the outgroup.

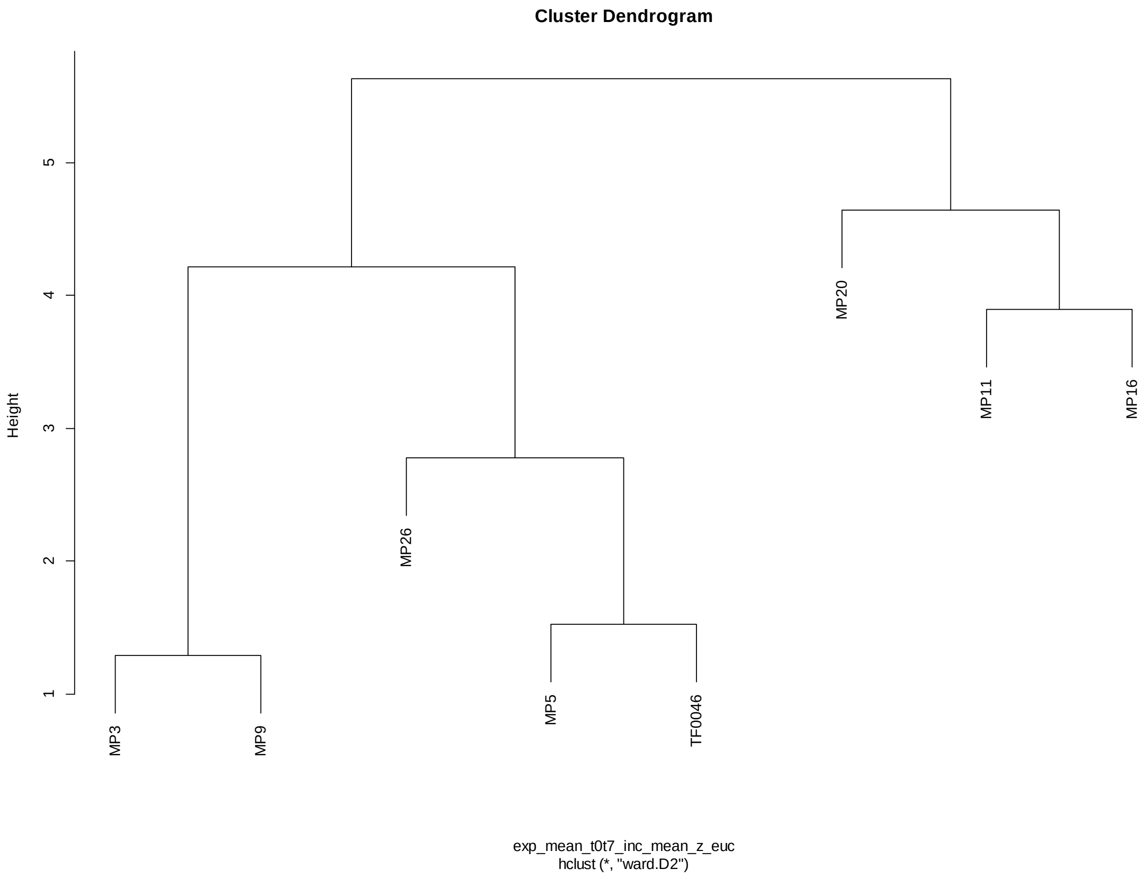

Supplementary Figure 6. Dendrogram based on hierarchical clustering of physico-chemical surface water properties, averaged from t_0 and t_7 parameters of the incubation water. Data was z-transformed and the Ward-method was used for clustering based on Euclidian distance.

Supplementary References

Letunic, I., and Bork, P. (2016). Interactive tree of life (iTOL) v3: An online tool for the display and annotation of phylogenetic and other trees. *Nucleic Acids Res.* 44, W242–W245. doi:10.1093/nar/gkw290.

Ludwig, W., Strunk, O., Westram, R., Richter, L., Meier, H., Kumar, Y., et al. (2004). ARB: a software environment for sequence data. *Nucleic Acids Res.* 32, 1363–1371. doi:10.1093/nar/gkh293.

Oksanen, J., Blanchet, F. G., Friendly, M., Kindt, R., Legendre, P., McGlinn, D., et al. (2018). *vegan: Community Ecology Package*. Available at: https://CRAN.R-project.org/package=vegan.

Pruesse, E., Peplies, J., and Glöckner, F. O. (2012). SINA: Accurate high-throughput multiple sequence alignment of ribosomal RNA genes. *Bioinformatics* 28, 1823–1829. doi:10.1093/bioinformatics/bts252.

R Core Team (2017). *R: A language and environment for statistical computing*. Vienna, Austria: R Foundation for Statistical Computing Available at: https://www.R-project.org/.
